# Genetic identification of inherited cystic kidney diseases for implementing precision medicine: a study protocol for a 3-year prospective multicenter cohort study

**DOI:** 10.1186/s12882-020-02207-8

**Published:** 2021-01-06

**Authors:** Hayne Cho Park, Hyunjin Ryu, Yong-Chul Kim, Curie Ahn, Kyu-Beck Lee, Yeong Hoon Kim, Yunmi Kim, Seungyeup Han, Yaerim Kim, Eun hui Bae, Seong Kwon Ma, Hee Gyung Kang, Yo Han Ahn, Eujin Park, Kyungjo Jeong, Jaewon Lee, Jungmin Choi, Kook-Hwan Oh, Yun Kyu Oh

**Affiliations:** 1 Department of Internal Medicine, Hallym University College of Medicine, Seoul, South Korea; 2 Department of Internal Medicine, Seoul National University College of Medicine, Seoul, South Korea; 3 Department of Internal Medicine, National Medical Center, Seoul, South Korea; 4 Department of Internal Medicine, Kangbuk Samsung Hospital, Seoul, South Korea; 5 Department of Internal Medicine, Busan Paik Hospital, Busan, South Korea; 6 Department of Internal Medicine, Keimyung University School of Medicine, Daegu, South Korea; 7 Department of Internal Medicine, Chonnam National University Medical School, Gwangju, South Korea; 8 Department of Pediatrics, Seoul National University Children’s Hospital, Seoul, South Korea; 9 Department of Pediatrics, Hallym University College of Medicine, Seoul, South Korea; 10 Department of Biomedical Sciences, Korea University College of Medicine, Seoul, South Korea; 11 Department of Internal Medicine, Seoul Metropolitan Government Seoul National University Boramae Medical Center, Seoul, South Korea

**Keywords:** Cohort study, Cystic kidney disease, High-throughput nucleotide sequencing, Genotype, Phenotype, Genetic association studies, Glomerular filtration rate

## Abstract

**Background:**

Inherited cystic kidney disease is a spectrum of disorders in which clusters of renal cysts develop as the result of genetic mutation. The exact methods and pipelines for defining genetic mutations of inherited cystic kidney disease are not clear at this point. This 3-year, prospective, multicenter, cohort study was designed to set up a cohort of Korean patients with inherited cystic kidney disease, establish a customized genetic analysis pipeline for each disease subtype, and identify modifying genes associated with the severity of the disease phenotype.

**Methods/design:**

From May 2020 to May 2022, we aim to recruit 800 patients and their family members to identify pathogenic mutations. Patients with more than 3 renal cysts in both kidneys are eligible to be enrolled. Cases of simple renal cysts and acquired cystic kidney disease that involve cyst formation as the result of renal failure will be excluded from this study. Demographic, laboratory, and imaging data as well as family pedigree will be collected at baseline. Renal function and changes in total kidney volume will be monitored during the follow-up period. Genetic identification of each case of inherited cystic kidney disease will be performed using a targeted gene panel of cystogenesis-related genes, whole exome sequencing (WES) and/or family segregation studies. Genotype-phenotype correlation analysis will be performed to elucidate the genetic effect on the severity of the disease phenotype.

**Discussion:**

This is the first nationwide cohort study on patients with inherited cystic kidney disease in Korea. We will build a multicenter cohort to describe the clinical characteristics of Korean patients with inherited cystic kidney disease, elucidate the genotype of each disease, and demonstrate the genetic effects on the severity of the disease phenotype.

**Trial registration:**

This cohort study was retrospectively registered at the Clinical Research Information Service (KCT0005580) operated by the Korean Center for Disease Control and Prevention on November 5th, 2020.

## Background

Inherited cystic kidney disease (iCKD) is a hereditary disorder in which clusters of cysts develop within the kidneys [1, 2]. Approximately 100 genes involved in renal cystogenesis are known to result in dysfunction of a hair-like organelle called the cilium [3]. Therefore, iCKD is otherwise called ciliopathy [4, 5]. iCKD encompasses autosomal-dominant polycystic kidney disease (ADPKD), tuberous sclerosis complex [6], von Hippel-Lindau disease, autosomal-dominant tubulointerstitial kidney disease (ADTKD) [7], and pediatric diseases such as autosomal recessive polycystic kidney disease (ARPKD) [8] and nephronophthisis (NPHP) [9]. There are still many other disorders in which causative mutations have not been found by molecular diagnosis.

Although iCKDs are caused by genetic derangement, most are diagnosed by clinical impressions other than molecular diagnosis. However, clinical diagnosis is not always easy because iCKDs often share common clinical manifestations. Therefore, genetic testing is important to establish a correct diagnosis and treatment. A recent report by Bullich et al. demonstrated that they could confirm diagnosis in 32% of cases with unspecified clinical diagnosis by establishing a kidney gene panel [10]. They also showed that genetic testing changed the clinical diagnosis in 2% of cases. Therefore, genetic testing should be the most important venue to confirm diagnosis and establish precision medicine.

Moreover, the exact methods and pipelines to find genetic mutations in iCKDs are not clear at this point. It may be reasonable to perform targeted exome sequencing or Sanger sequencing of *PKD1* and *PKD2* to define pathogenic mutations in well-known clinical phenotypes such as ADPKD. However, our previous study demonstrated that approximately 20% of patients with typical ADPKD did not reveal causative germline mutations by targeted exome sequencing of *PKD1* and *PKD2* [11]. In addition, extrarenal manifestations often do not follow renal manifestations. For example, the severity of polycystic liver accompanying ADPKD does not always correlate with the severity of renal disease [12]. Therefore, modifying genetic effects or gene dosage effects may play a role in determining the severity of renal and extrarenal phenotypes in ADPKD [13]. In addition, apart from typical ADPKD, there are patients with atypical polycystic kidney disease who either do not show concordant features within the family, do not have typical imaging features of ADPKD, or have discordant disease severity between renal volume and renal function [14]. Mutations in *GANAB* and *DNAJB11* are known to cause a mild phenotype of polycystic kidney and liver disease [15, 16]. However, the exact prevalence and prognosis of atypical polycystic kidney disease is unknown at this point. Last, iCKDs in the pediatric population are typically rare diseases, and their molecular diagnoses are even more difficult. Therefore, building a cohort of iCKDs is necessary to reveal their genetic characteristics.

Therefore, we designed a 3-year prospective, multicenter, cohort study to establish a cohort of Korean iCKD patients, establish a customized genetic analysis pipeline that can genotype each iCKD and identify the modifying genes associated with the severity of the disease phenotype.

## Methods/design

### Study design and settings

This is a 3-year prospective, multicenter, cohort study to elucidate genotype-phenotype associations among iCKD patients. A total of 11 medical centers from 9 tertiary hospitals in Korea will participate in this study. Seven centers will enroll and collect data from adult patients, and 4 centers will enroll pediatric patients. We established a research team, statistical analysis team, database team, sequencing and biobanking team, and genetic analysis team to perform this large nationwide project. The research team is composed of 26 clinicians and 15 clinical research coordinators from 11 medical centers. The role of the research team is to recruit eligible patients and collect clinical data. The statistical analysis team supports the calculation of sample size, distribution of enrollment according to iCKD subclasses, and statistical analysis. The database team collects clinical and genetic data from each patient and builds an electronic case report form to store and manage the dataset. The database team also performs imaging analysis quantitatively and qualitatively from nonenhanced computed tomography (CT) or sonography. The team for sequencing and biobanking is outsourced to Macrogen, Inc. to collect whole blood from each medical center and perform initial genetic analysis to produce sequencing data. The residual DNA samples will be prepared for biobanking after quantity and quality checks. Finally, the genetic analysis team is composed of bioinformaticians to interpret the results of sequencing data and to determine the pathogenicity of each variant.

### Study participants

A total of 800 participants are planned to be enrolled from May 19, 2019 to May 18, 2022. Patients with ≥3 renal cysts in both kidneys are eligible to be enrolled. Those who are not able to give informed consent or are pregnant will be excluded from enrollment. Cases of simple renal cysts and acquired cystic kidney disease that involve cyst formation as the result of renal failure will also be excluded from this study. However, patients with end-stage kidney disease who are receiving renal replacement therapy due to iCKD can be enrolled.

The patients will be classified into typical ADPKD, atypical polycystic kidney disease, and other iCKDs after enrollment. Typical ADPKD is defined according to Pei-Ravine criteria as previously described [17]. Atypical polycystic kidney disease is defined either when the case is typical ADPKD but the patient does not have a family history of polycystic kidney disease or when the imaging phenotype is atypical as follows: unilateral, asymmetric, segmental, lopsided, bilateral or unilateral atrophic kidneys [18]. Other iCKDs are rare disease entities in children and adolescents. The other iCKDs include but are not limited to the tuberous sclerosis complex, von Hippel-Lindau disease, ADTKD, ARPKD, *HNF-1β*-related disease and NPHP. Among the 800 participants, approximately 650 patients with typical ADPKD, 90 patients with atypical polycystic kidney disease, and 60 patients with other iCKDs will be enrolled.

The parents, siblings, or children of the enrolled patients are recommended to participate in the study by giving whole blood samples for a segregation study. We will collect family samples when genetic diagnosis is undetermined, genotype-phenotype severity is not matched, and when the extrarenal manifestation is severe. We will also collect family samples from the families with more than 3 affected individuals to define gene penetrance and modify gene effects.

### Data collection at enrollment

Demographic data, including age, sex, height and weight, will be collected. The age of diagnosis of iCKD and associated symptoms at initial diagnosis will be collected. Medical history of diabetes, hypertension, cardiovascular disease, and stroke will be investigated. Family history of iCKD, diabetes, hypertension, chronic kidney disease, dialysis, and death will be evaluated. In particular, a genetic tree will be drawn upon enrollment including 3 generations (affected and unaffected individuals). The presence of renal and extrarenal complications and their types will be recorded. Medication data, including on antihypertensive drugs and glucose-lowering therapy, will be collected. Blood pressure will be checked upon enrollment in the office. All patients will be asked to fill out the following questionnaires upon enrollment: 5 Level version of European Quality of Life 5 Dimensions questionnaire (EQ-5D-5L, adult subjects) and Pediatric Quality of Life Inventory TM (PedsQL 4.0 Generic Core Scales, pediatric subjects) to assess the quality of life of the affected patients, Patient Health Questionnaire-9 (PHQ-9) to evaluate depressive symptoms, and the modified Subjective Global Assessment (mSGA) to assess the nutritional status of the subjects.

Laboratory assessment included complete blood cell counts (white blood cells, hemoglobin, platelets), blood urea nitrogen and serum creatinine, total calcium and phosphorus, serum sodium, potassium, chloride, total carbon dioxide, total cholesterol, serum albumin, uric acid, highly sensitive C-reactive protein, urinalysis with microscopy, spot urine protein to creatinine ratio, random urine uric acid, calcium, phosphorus, sodium, potassium, chloride, and osmolality. Genetic samples will be collected once during the study period for genetic analysis. Approximately 18 mL of whole blood will be collected in 3 EDTA bottles for each adult participant, 6 mL for each family member and 4–5 mL for each child participant. The collected blood samples will be refrigerated at 4 °C until delivery to the sequencing company. The sequencing company will extract DNA from the whole blood and aliquots in several tubes to store at − 70 °C before sequencing or biobanking.

Kidney imaging will be performed at enrollment. If the patients have already undergone imaging studies within 1 year, the patients can undergo other imaging studies within a 2-year interval. Adult patients will undergo a nonenhanced kidney CT, and children and adolescents will undergo kidney sonography.

### Data collection, monitoring, and follow up

The total study scheme and annual assessment plan are depicted in Table 1. Annual laboratory assessment will be performed after enrollment for 2 years. The laboratory assessment includes the complete blood cell counts, blood urea nitrogen and serum creatinine, serum calcium and phosphorus, serum uric acid, urinalysis with microscopy, and spot urine protein to creatinine ratio. Kidney imaging will be performed every 2 years to calculate the rate of total kidney volume growth.

**Table 1:** Study schedule

Parameter	Screen	B0	1y	2y
Informed consent	v			
Demographic information	v			
Medical history	v			
Eligibility confirmation	v			
Recent events			v	v
Medications		v	v	v
Quality of life questionnaires (EQ-5D-5L (adult), PedsQL 4.0 Generic Core Scales (pediatric))		v		
Depression assessment (PHQ-9)		v		
Nutritional assessment (mSGA)		v		
Systolic and diastolic blood pressure		v	v	v
Complete blood cell count (white blood cells, hemoglobin, platelet count, hematocrit, reticulocyte count, neutrophil count)		v	v	v
Serum calcium/phosphorus		v	v	v
Serum uric acid		v	v	v
Serum protein/albumin		v	v	v
Serum blood urea nitrogen/creatinine/estimated glomerular filtration rate		v	v	v
Plasma sodium/potassium/chloride/total carbon dioxide		v	v	v
Serum total cholesterol		v		
Highly sensitive C-reactive protein		v		
Urinalysis with microscopy		v	v	v
Spot urine protein/creatinine ratio		v	v	v
Spot urine uric acid		v		
Spot urine sodium/potassium/chloride		v		
Spot urine calcium/phosphorus		v		
Spot urine osmolality		v		
Kidney CT (adult) or abdomen sonography (pediatric)		v		

*Abbreviations*: *CT* Computed tomography, *PedsQL* Pediatric Quality of Life inventory, *PHQ-9* Patient Health Questionnaire-9, *mSGA* Modified Subjective Global Assessment

Electronic case report forms will be developed, including demographic sheets, laboratory assessments, volumetry, and genetic analysis information. The electronic case report form will be opened to the participating researchers and clinical research coordinators to fill out and modify patient information. A family tree will be drawn and stored in electronic case report form by scanning the sheet.

### Evaluation of renal function

Renal function will be evaluated upon enrollment and every year thereafter. For the adult patients, renal function will be measured using the estimated glomerular filtration rate calculated by the Chronic Kidney Disease Epidemiology Collaboration (CKD-EPI) equation [19]. If the patients are in end-stage renal disease or receive renal replacement therapy upon enrollment, renal function will be evaluated retrospectively to calculate the renal function decline rate. For children, renal function will be measured using the estimated glomerular filtration rate calculated by the Schwartz equation [20].

### Imaging analysis

The patients will be classified into typical and atypical cystic kidney disease. Nonenhanced kidney CT will be performed in adult patients. The patients will be encouraged to take water before the CT exams to accurately distinguish the liver and stomach anatomically. All image files from the nonenhanced CT will be retrieved to a workstation and inspected to confirm complete coverage of both kidneys and liver. Images will be reconstructed into 5 mm sections in axial images and 3 mm sections in both coronal and sagittal sections before volume measurements. The total kidney volume will be measured by a professionally educated radiologist. Total kidney volume will be measured by 2 methods: the stereologic method by using semiautomatic volumetry software (ImageJ version 1.5a, https://imagej.nih.gov/ij/) [21] and the manual method by using the Mayo ellipsoid method [18, 22]. Expanded imaging classification will be applied to typical and atypical polycystic kidney cases [23]. The sonographic images and their interpretations will be collected from pediatric patients. The number and distribution of cysts and their characteristics will be reported in our case report form. The radiologist will also measure the muscle area to assess the nutritional status as previously reported [24].

### Genetic pipeline

**Fig. 1:**
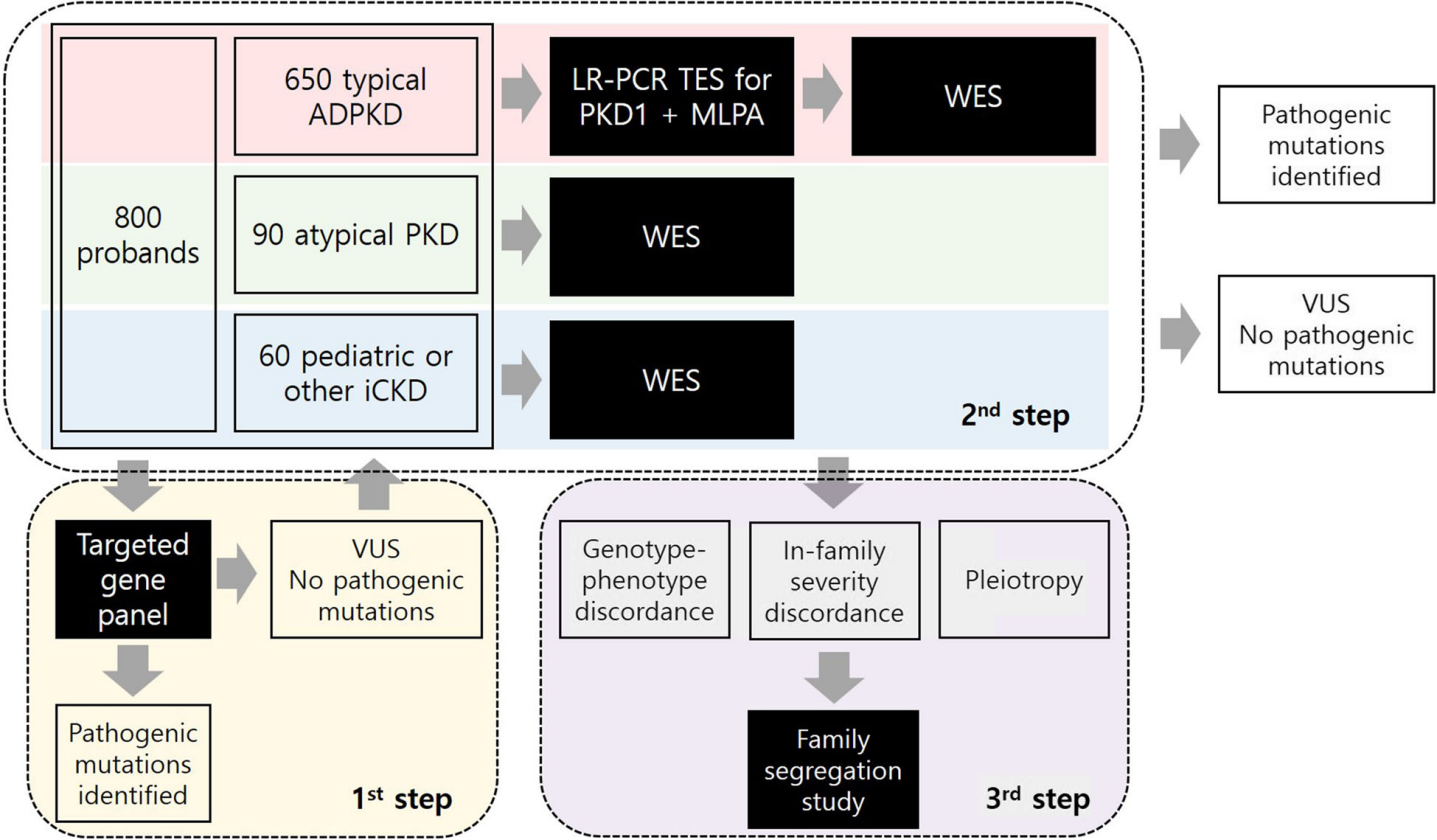
Genetic analysis pipeline. A total of 800 probands with iCKD will be enrolled in the study. They will be classified into typical ADPKD, atypical polycystic kidney disease, or other/pediatric iCKDs. For the first screening genetic test, a targeted gene panel of 89 cytogenesis-related genes will be applied to the total population. All the variants will be analyzed by bioinformaticians to identify pathogenic mutations. For those with variants of undetermined significance (VUS) or no variants found by the gene panel, different genetic approaches will be taken for each class of iCKD. For those with typical ADPKD, targeted exome sequencing of *PKD1* after long-range PCR combined with MLPA will be performed to identify pathogenic mutations. If the mutations are not found by this method, WES will take place. For those with atypical polycystic kidney disease or pediatric iCKD, WES will be performed to identify pathogenic mutations. For the last step of genetic diagnosis, a family segregation study will be performed to elucidate the cause of genotype-phenotype discordance, in-family severity discordance, or discordance between renal and extrarenal manifestations. Abbreviations: ADPKD, autosomal dominant polycystic kidney disease; iCKD, inherited cystic kidney disease; PCR, polymerase chain reaction; MLPA, multiplex ligation-dependent probe amplification; PKD, polycystic kidney disease; VUS, variant of undetermined significance; WES, whole exome sequencing

We will use a stepwise approach to confirm genetic diagnosis. First, we will use a targeted gene panel for the screening method. We designed a targeted gene panel (Twist Bioscience, San Francisco, CA, USA) encompassing 0.5 megabases, including 89 genes related to cystogenesis or ciliopathy as well as genes that are associated with extrarenal phenotypes such as liver cysts (Table 2). Twist technology has provided high-quality target enrichment probes to cover target genes uniformly and efficiently [25]. Targeted exon capture will be performed on genomic DNA samples using a Twist custom panel kit followed by 101 base paired-end sequencing on an Illumina NovaSeq6000 platform (Illumina, San Diego, CA, USA). Sequence reads will be aligned to the human reference genome (GRCh37/hg19) using BWA-MEM and further processed to call single nucleotide variants and indels following the GATK Best Practices workflow [26]. All variants covered by independent sequence reads with a depth of 8x or greater will be annotated with ANNOVAR. All variants will be visualized in silico to eliminate false positives. Additional genetic testing will be performed if the pathogenic mutations were not found using a targeted gene panel or if the patients have severe renal or extrarenal phenotypes compared to other family members. If the patients are clinically classified as having typical ADPKD but pathogenic mutations are not found using a targeted gene panel, the patients will undergo targeted resequencing with long-range polymerase chain reaction (PCR) combined with multiplex ligation-dependent probe amplification (MLPA) to detect large deletions. WES will take place in the following cases: 1) if the patients are clinically classified as typical ADPKD but no pathogenic mutations are found using a previous method, 2) if the patients are clinically classified as atypical polycystic kidney disease or other iCKD but pathogenic mutations are not found using a targeted gene panel, 3) if the patients present with a severe phenotype and variants cannot explain the severity, or 4) if the patients show extremely different phenotypes compared to other family members. Familial segregation analysis will also take place to define the pathogenicity of variants. A schematic representation of the genetic workflow is shown in Fig. 1.

**Table 2:** Coverage of cystogenesis-related targeted gene panel

Target gene	Previously reported disease	Coverage
*AHI1*	Joubert syndrome	100%
*ALG8*	Polycystic liver disease	88.61%
*ARL13B*	Joubert syndrome	*98.72%*
*ATF6B*	ER candidate gene (polycystic liver)	100%
*ATXN3*	ER candidate gene (polycystic liver)	97.97%
*AVP*	Polycystic kidney disease	100%
*AVPR2*	Polycystic kidney disease	100%
*C5ORF42*	Joubert syndrome	100%
*CAPN2*	ER candidate gene (polycystic liver)	99.1%
*CC2D2A*	Joubert syndrome	98.13%
*CEP120*	Joubert syndrome	97.02%
*CEP164*	NPHP	100%
*CEP290*	NPHP/MKS	100%
*COL4A1*	Hereditary angiopathy with nephropathy, aneurysms, and muscle cramps	100%
*COL4A3*	Alport syndrome	99.29%
*COL4A4*	Alport syndrome	100%
*COL4A5*	Alport syndrome	100%
*CSPP1*	Joubert syndrome	97.3%
*CYS1*	Cilia-associated cystic genes	100%
*DNAJB11*	Atypical polycystic kidney disease	100%
*DYNC2H1*	Cilia-associated cystic genes	100%
*EDEM3*	ER candidate gene (polycystic liver)	100%
*EYA1*	Branchiootorenal dysplasia syndrome	100%
*FAN1*	Karyomegalic interstitial nephritis	100%
*GANAB*	ADPKD	100%
*GLIS2*	NPHP	100%
*GLIS3*	Neonatal diabetes, hypothyroidism, and cystic kidney disease	100%
*HNF1B*	Renal cysts and diabetes syndrome	100%
*HSP90AA1*	ER candidate gene (polycystic liver)	100%
*HSPA6*	ER candidate gene (polycystic liver)	100%
*HYOU1*	ER candidate gene (polycystic liver)	100%
*IFT140*	Cilia-associated cystic genes	100%
*IFT172*	Cilia-associated cystic genes	100%
*IFT80*	Cilia-associated cystic genes	99.58%
*IFT88*	Cilia-associated cystic genes, phenotype resembling ADPKD	98.7%
*INPP5E*	Joubert syndrome	100%
*INVS*	NPHP	100%
*IQCB1*	NPHP	100%
*KIAA0586*	Joubert syndrome	93.79%
*LRP5*	Polycystic liver disease	100%
*MKS1*	Joubert syndrome/MKS	100%
*MUC1*	ADTKD	100%
*NEK1*	Polycystic kidney disease	100%
*NEK8*	NPHP	100%
*NGLY1*	ER candidate gene (polycystic liver)	100%
*NPHP3*	NPHP	98.29%
*NPHP4*	NPHP	100%
*OFD1*	OFD	100%
*PARK2*	ER candidate gene (polycystic liver)	100%
*PAX2*	Optic nerve coloboma, renal hypoplasia	100%
*PKD1*	ADPKD	100%
*PKD2*	ADPKD	100%
*PKHD1*	ARPKD	100%
*PMM2*	Polycystic kidney disease with hyperinsulinemic hypoglycemia	100%
*PRKCSH*	Polycystic liver disease	100%
*REN*	Familial hyperproreninemia, high blood pressure	100%
*RPGRIP1L*	Joubert syndrome/MKS	96.64%
*SDCCAG8*	NPHP	94.23%
*SEC24B*	ER candidate gene (polycystic liver)	97.43%
*SEC24C*	ER candidate gene (polycystic liver)	100%
*SEC24D*	ER candidate gene (polycystic liver)	100%
*SEC31A*	ER candidate gene (polycystic liver)	97.42%
*SEC31B*	ER candidate gene (polycystic liver)	100%
*SEC61A1*	ER candidate gene (polycystic liver)	100%
*SEC61A2*	ER candidate gene (polycystic liver)	100%
*SEC61B*	Polycystic liver disease	100%
*SEC62*	ER candidate gene (polycystic liver)	100%
*SEC63*	Polycystic liver disease	95.11%
*TCTN2*	Joubert syndrome	100%
*TMEM216*	Joubert syndrome	100%
*TMEM67*	NPHP/Joubert syndrome/MKS	94.94%
*TSC1*	Tuberous sclerosis complex	100%
*TSC2*	Tuberous sclerosis complex	100%
*TTC21B*	NPHP	100%
*TXNDC5*	ER candidate gene (polycystic liver)	95.15%
*UBE4B*	ER candidate gene (polycystic liver)	100%
*UGGT1*	ER candidate gene (polycystic liver)	100%
*UGGT2*	ER candidate gene (polycystic liver)	100%
*UMOD*	ADTKD	100%
*VHL*	Von Hippel–Lindau syndrome	100%
*WDR19*	NPHP	100%
*WDR34*	Cilia-associated cystic genes	100%
*WDR35*	Cilia-associated cystic genes	100%
*WDR60*	Cilia-associated cystic genes	100%
*WFS1*	ER candidate gene (polycystic liver)	100%
*XBP1*	Polycystic kidney and liver diseases	100%

*Abbreviations*: *ADPKD* Autosomal dominant polycystic kidney disease, *ADTKD* Autosomal dominant tubulointerstitial kidney disease, *ARPKD* Autosomal recessive polycystic kidney disease, *ER* Endoplasmic reticulum, mks Meckel syndrome, *NPHP* Nephronophthisis, *OFD* Orofaciodigital syndrome

### Genotype-phenotype correlation analysis

Statistical analyses will be performed using a recent version of SPSS software (IBM Corp., Armonk, NY, USA). A linear regression model will be performed to identify the correlation between genotype and clinical parameters, including total kidney volume and estimated glomerular filtration rate. The cases will be classified into 5 classes using the Mayo imaging classification before analysis. The cases will also be classified into 6 groups according to the chronic kidney disease stages. Analysis of covariance, the Mann-Whitney test, and the chi-square test will be performed to compare variables between groups. The modifier effect of multiple genes on renal and extrarenal manifestations will also be assessed. A *P* value < 0.05 will be considered statistically significant.

## Discussion

This study is the first prospective, multicenter cohort study that will evaluate the genetic profiles and their clinical correlation among patients with iCKDs in Korea. There are some international cohorts to define the genetic characteristics of various cystic kidney diseases and their association with phenotypes. The Network for Early Onset Cystic Kidney Disease (NEOCYST) is a government-funded multicenter network that collects clinical and genetic data to understand the underlying pathogenesis of hereditary cystic kidney disease [27]. The Consortium for Radiologic Imaging Study of Polycystic kidney disease (CRISP) group prospectively collected clinical, radiological, and genetic data to perform genotype-phenotype studies [28, 29]. The Toronto Genetic Epidemiology Study of Polycystic kidney disease (TGESP) also examined the prevalence of different mutation classes and their association with phenotypes [30]. In Korea, there have only been single-center driven cohort studies for specific diseases. However, there has not been a nationwide multicenter iCKD network to collect epidemiologic, clinical, radiological, and genetic data prospectively. This multicenter iCKD cohort will establish a concrete database and biobank of the Korean iCKD population from which genotype-phenotype association studies can be performed.

Since the clinical diagnosis of iCKD is not always easy, genetic characterization will help to confirm the diagnosis of each iCKD case and to elucidate the heterogeneity of disease manifestations within the family. Since there are over 100 genes that can result in ciliopathies, Sanger sequencing or targeted exome sequencing of a few genes can be time-consuming and costly. The targeted gene panel approach through parallel sequencing of targeted subsets of disease-specific genes may be an effective screening method for iCKD cases. Recent papers have also reported the effectiveness of gene panels and subsequent WES approaches in confirming genetic diagnosis [10, 31, 32]. Therefore, we designed a targeted gene panel for the initial screening method to find causal variants. The gene panel can be designed and customized for research purposes. We included 13 genes associated with Joubert syndrome, 27 genes associated with polycystic liver, 8 genes associated with ADPKD, 1 gene associated with ARPKD, 11 genes associated with NPHP, 3 genes associated with Alport syndrome, 2 genes associated with ADTKD, 2 genes associated with tuberous sclerosis complex and 19 other ciliopathy-related genes in our targeted gene panel. The composition of the gene panel will help us not only identify causal variants for renal cystic disease but also explain the heterogeneity of extrarenal manifestations in the same disease.

Patient recruitment from secondary and tertiary hospitals across the country will represent the Korean cohort of iCKDs. The sample size of 800 should provide sufficient statistical power to address the heterogeneity of typical ADPKD, atypical polycystic kidney disease, and pediatric iCKDs. In particular, the establishment of a pediatric iCKD subcohort and atypical polycystic kidney disease cohort can be helpful in defining pathogenic mutations in each group because they are so rare, and genetic diagnosis of each case can be difficult without building a multicenter cohort. The five well-organized study teams (research team, statistical analysis team, database team, sequencing and biobanking team, and genetic analysis team) of this study will facilitate the study process. Various other factors, such as central electronic case report forms, researcher meetings, study nurse meetings, comprehensive study analyses and regular monitoring, will keep the quality of this study as high as possible.

Potential limitations include the observational nature of the study and short duration of follow-up. Although we will recruit approximately 15% of the total iCKD population in Korea, we cannot exclude potential selection bias since most of the patients will be recruited through secondary and tertiary hospitals.

In summary, we will establish a prospective genetic cohort of iCKDs in Korea with 800 pedigrees in which we collect demographic and clinical data as well as family tree and laboratory follow-up data. We will establish a genetic pipeline in typical ADPKD, atypical polycystic kidney disease, and pediatric iCKD cohorts and analyze genotype-phenotype correlations in renal and extrarenal manifestations. This study will help us implement precision medicine for Korean iCKD patients.

## Data Availability

Not applicable.

## Abbreviations

ADPKD: Autosomal dominant polycystic kidney disease
ADTKD: Autosomal dominant tubulointerstitial kidney disease
ARPKD: Autosomal recessive polycystic kidney disease
CKD-EPI: Chronic Kidney Disease Epidemiology Collaboration
CRISP: Consortium for Radiologic Imaging Study of Polycystic Kidney Disease
CT: Computed tomography
EQ-5D-5L: 5 Level version of European Quality of life 5 Dimensions questionnaire
ER: Endoplasmic reticulum
iCKD: Inherited cystic kidney disease
MKS: Meckel syndrome
MLPA: Multiplex ligation-dependent probe amplification
mSGA: Modified Subjective Global Assessment
NEOCYST: Network for Early Onset Cystic Kidney Disease
NPHP: Nephronophthisis
OFD: Orofaciodigital syndrome
PCR: Polymerase chain reaction
PedsQL: Pediatric Quality of Life inventory
PHQ-9: Patient Health Questionnaire-9
TGESP: Toronto Genetic Epidemiology Study of Polycystic kidney disease
VUS: Variant of undetermined significance
WES: Whole exome sequencing
